# The Role of Child Health Days in the Attainment of Global Deworming Coverage Targets among Preschool-Age Children

**DOI:** 10.1371/journal.pntd.0004206

**Published:** 2015-11-06

**Authors:** Richard Senam Kumapley, Roland Kupka, Nita Dalmiya

**Affiliations:** 1 Micronutrients Unit, Nutrition Section, UNICEF Headquarters, New York, New York, United States of America; 2 Nutrition Section, UNICEF Regional Office for West and Central Africa, Dakar, Senegal; PATH, UNITED STATES

## Abstract

**Background:**

Global deworming programs aim to reach 75% of at-risk preschool-age children (pre-SAC) by 2020. The 2013 global pre-SAC deworming coverage initially published by the World Health Organization (WHO) was 23.9%, but this estimate inadequately captured deworming delivered through Child Health Day (CHD) platforms.

**Objective:**

To update global and regional coverage estimates of pre-SAC deworming in 2013 by supplementing data from the WHO Preventive Chemotherapy and Transmission Control (PCT) databank with national CHD data.

**Methods:**

UNICEF country offices (n = 82) were mailed a questionnaire in July 2014 to report on official national biannual CHD deworming coverage as part of the global vitamin A supplementation coverage reporting mechanism. Coverage data obtained were validated and considered for inclusion in the PCT databank in a collaboration between UNICEF and WHO. Descriptive statistical analyses were conducted to update the number of pre-SAC reached and the number of treatments delivered.

**Results:**

Of the 47 countries that responded to the UNICEF pre-SAC deworming questionnaire, 73 data points from 39 countries were considered for inclusion into the WHO PCT databank. Of these, 21 new data points were from 12 countries were newly integrated into the WHO database. With this integration, deworming coverage among pre-SAC increased to 49.1%, representing an increase in the number of children reached and treatments administered from 63.7 million to 130.7 million and 94.7 million to 234.8 million, respectively. The updated databank comprised 98 mass deworming activities conducted in 55 countries, in which 80.4% of the global pre-SAC population requiring deworming reside. In all, 57 countries requiring deworming were not yet represented in the database.

**Conclusions:**

With the inclusion of CHD data, global deworming programs are on track to achieving global pre-SAC coverage targets. However, further efforts are needed to improve pre-SAC coverage reporting as well as to sustain and expand deworming delivery through CHDs and other platforms.

## Author Summary

Soil-transmitted helminthiases are a group of parasitic diseases caused by intestinal worms that are linked to poor physical and cognitive development among preschool aged children. The administration of deworming drugs designed to reduce the intensity of the worm infection in the child is effective and efficient intervention to control the disease and has set the goal of deworming 75% of at-risk children by 2020. However, global WHO-reported coverage decreased from 37.1% to 24.7% from 2010 to 2012. In 2013, the first coverage estimate released was 23.9%, but as in previous years, this estimate did not adequately capture coverage achieved through Child Health Days, which are integrated campaign-style events where deworming is often co-delivered alongside vitamin A supplementation and other high impact child interventions. In this paper, we mailed a questionnaire to UNICEF country offices requesting data pertaining to preschool age deworming conducted through Child Health Days. After reviewing submissions and integrating data into the global databank, we report that the global coverage now stands at 49.4% putting us on track to achieve the global goal by 2020. The sharp increase in coverage illustrates the importance of Child Health Days for attaining global pre-SAC coverage goals.

## Introduction

Soil-transmitted helminthiases (STH) are a group of parasitic diseases caused by intestinal worms that are transmitted to humans through fecally-contaminated soil [1]. Although STH is classified as a neglected tropical disease (NTD), it is estimated that over 3.5 billion people are at risk of infection, 10%-15% of whom are children of preschool age (pre-SAC) [2]. STH-related morbidity increases with higher worm load and includes iron deficiency, protein malnutrition and poor cognitive development [3, 4]. The regular deworming of pre-SAC with anthelminthic agents is an efficient and effective method of keeping STH worm loads below the levels associated with morbidity [1, 5].

In its strategic plan on “Eliminating Soil-Transmitted Helminths as a Public Health Problem in Children”, the World Health Organization (WHO) outlined strategies and milestones towards “eliminating [STH-related] morbidity in all children by 2020” [6]. The plan aims to increase global pre-SAC deworming coverage to 50% by 2015 and 75% by 2020 [6]. The Preventive Chemotherapy and Transmission Control (PCT) databank was instituted to track progress to global coverage targets by combining coverage information from different sources [7]. According to the databank, global pre-SAC deworming coverage has progressively declined from 37.1% in 2010 to 30.6% in 2011 and 24.7% in 2012 [8–10]. The initial coverage estimate for 2013 was 23.9% [11].

The initial 2013 coverage estimates were generated from 43 countries representing 54.0% of the global pre-SAC population requiring deworming [11, 12]. WHO and its partners recognize that not all countries requiring deworming report data to the databank and that not all mass deworming administrations (MDAs) are captured among reporting countries [13]. There has been no systematic attempt to collect data on deworming administered through Child Health Days, which are common delivery mechanisms for deworming alongside vitamin A supplementation (VAS) and other high-impact interventions among pre-SAC [14]. This paper describes the first systematic attempt to collect CHD deworming coverage in an effort to update the global 2013 pre-SAC coverage figures in the global WHO PCT databank.

## Method

### Collection and Review of UNICEF Deworming Coverage Data

UNICEF developed a spreadsheet-based questionnaire for deworming coverage data that was developed based on the VAS CHD reporting form used for official global VAS reporting [15]. The form includes questions on deworming coverage by age group, frequency, type of anthelminthic used and distribution mechanism employed. In line with the VAS reporting methodology, data on MDA were collected separately for semester 1 (January–June, 2013) and semester 2 (July–December, 2013). Where possible, the PCT databank has country entries separated by rounds, we refer to each entry in the PCT databank as a data point [7]. In July 2014, UNICEF Headquarters (HQ) sent these forms to its 82 country programs prioritized for VAS programs [16]. In these countries, UNICEF staff were requested to complete these forms with implementing partners and to obtain approval from relevant government ministries. Upon receipt, UNICEF HQ reviewed the data for completeness and consistency. Preliminary data or data that had not been officially approved by the government were excluded from further analysis.

### The WHO PCT Databank

The WHO PCT Databank is the official global tracking tool for global pre-SAC deworming coverage [7]. Started in 2003, it contains country-specific data provided by national NTD focal points, the Schistosomiasis Control Initiative, and development partners [7, 12]. Each record in the database presents the number of pre-SAC that require deworming, the program target population, the program coverage (the proportion of individuals treated as per program target set) and the national coverage (the proportion of the population requiring deworming in the country that has been treated). This database is continuously updated. WHO initially published the 2013 global pre-SAC coverage in January 15^th^ 2015 [11]. These estimates did not include the 2013 UNICEF CHD data.

### Data Merge

The coverage estimates collected by UNICEF were considered for updating the initial 2013 global coverage data in the PCT databank. As a first step, the semester-specific CHD events (i.e., data points) were compared with the data points already included in PCT database. All data points were then categorized into three groups: data points only present in the UNICEF deworming database, data points only present in the PCT databank and data points present in both databases. Data points only present in the UNICEF deworming database were validated based on number of pre-SAC treated, number of pre-SAC targeted and reported program coverage. The sources used as the standard for comparison were the latest available census and 2013 United Nations Population Division (UNPD) estimates [17]. Data points present in both databases were assessed for consistency and validation. They were considered to be identical if reported coverage was within 5%, in which case the WHO PCT data point was selected. In cases where data points were not considered identical, the data point containing the higher number of pre-SAC treated was chosen in line with WHO procedures. Any new or revised data points were uploaded into the WHO PCT databank and published [12].

### Calculating Coverage Estimates

The updated PCT databank was used to generate new global, regional and national level pre-SAC deworming coverage estimates. Regional estimates were calculated using WHO regional classifications [18]. These coverage estimates were defined as the proportion of the pre-SAC population requiring deworming that received treatment [10]. The number of pre-SAC that require deworming are determined by WHO in partnership with Ministries of Health (MoHs) after taking into account demographic, epidemiologic and sanitation data [19]. In addition, data reporting gaps were calculated by comparing the proportion of pre-SAC population requiring deworming for the responding countries against the global pre-SAC population requiring deworming over for the years 2006–2013 using both the initial and updated datasets. Standard descriptive statistical analyses were used to calculate the number of treatments provided by anthelminthic agent and to determine the number of MDAs that surpassed the program and national coverage targets of 75%. All analyses were conducted using Microsoft Excel (Microsoft, Redmond, WA).

## Results

**Fig 1:**
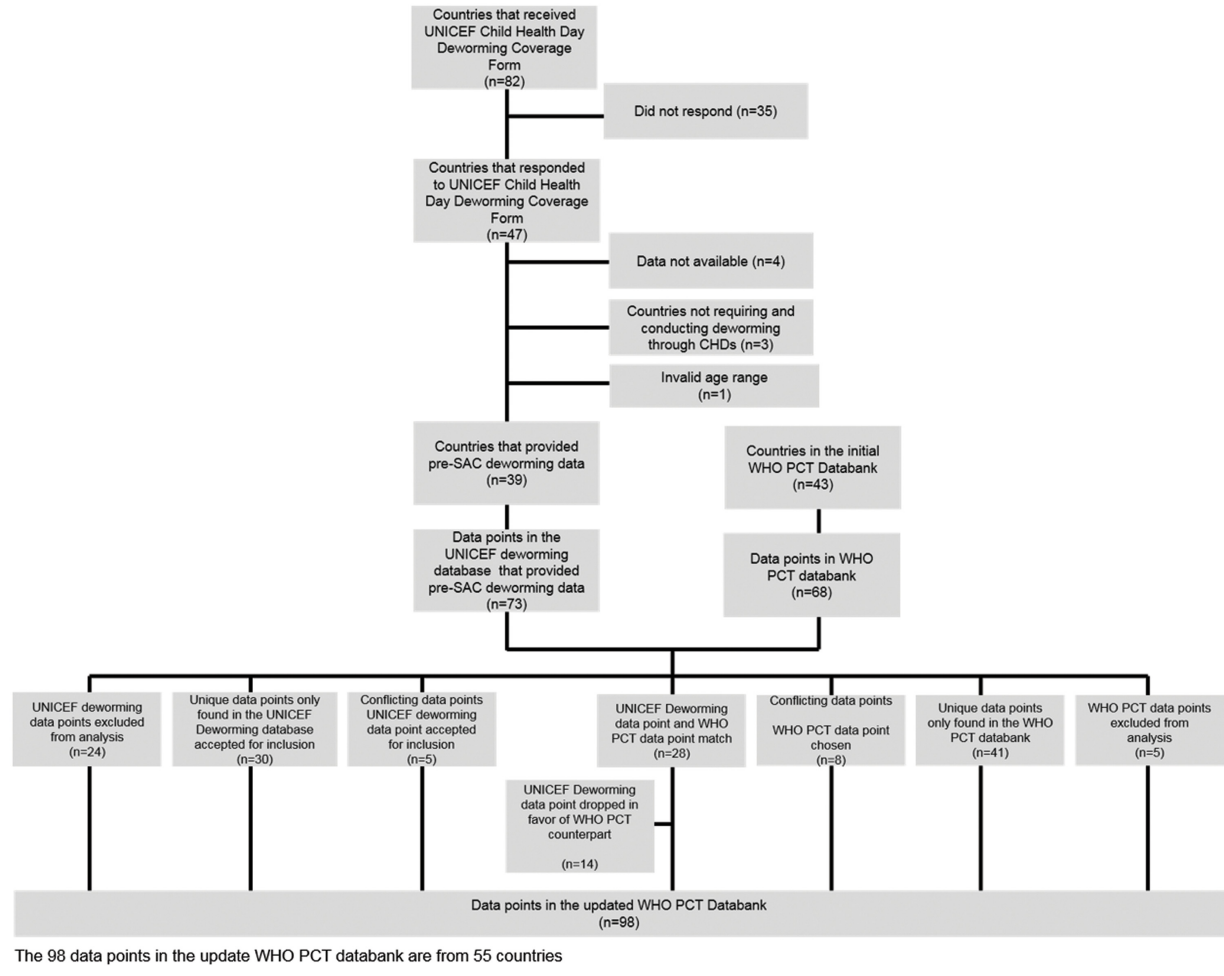
Integration of CHD data into the PCT databank. Data flowchart on the integration of Child Health Day data collected by UNICEF into the World Health Organization Preventive Chemotherapy and Transmission Control Databank, 2013. CHD, Child Health Day, Pre-SAC, preschool-age children, PCT, Preventive Chemotherapy and Transmission Control Databank,

Of the 82 UNICEF offices contacted, 47 (57%) submitted responses (Fig 1). Of these, a total of 39 countries had valid and nationally endorsed data points. In all, 27 (73%) were from Africa, 6 (16%) from South-East Asia, 3 (8%) from the Western Pacific region, 2 (5%) from East Mediterranean Region, and 1 (3%) from the Americas. The nationally validated datasets covered 74.9% of the global pre-SAC population requiring deworming. In total, the database contained 73 data points from the 39 respondent countries.

The version of the PCT databank used for the initial analysis contained 68 deworming data points from 43 of the 106 countries where deworming is required for STH (Table 1). In total, these countries represented 54% of the global pre-SAC population requiring deworming.

**Table 1:** Global gaps in deworming coverage reporting in the World Health Organization Preventive Chemotherapy and Transmission Control Databank, 2013.

		Initial Database^2^			Updated Database^3^		
Regions^1^	Number of Countries where deworming is required	Number of Countries	Proportion of pre-SAC population requiring deworming (%)	Data Gap^4^ (%)	Number of Countries	Proportion of pre-SAC population requiring deworming (%)	Data Gap^4^ (%)
Africa	43	16	19.5	80.5	26	76.8	23.2
The Americas	24	8	58.8	41.2	8	58.8	41.2
East Mediterranean	8	1	0.0	100	2	40.2	59.8
Europe	8	1	12.3	87.7	1	12.3	87.7
South-East Asia	8	7	99.8	0.2	8	99.9	0.1
Western Pacific	15	10	64.2	35.8	10	64.2	35.8
Global	106	43	54.0	46.0	55	80.4	19.6

Pre-SAC, preschool-age children.

^1^ WHO Regions

^2^ Initial Database, WHO Preventive Chemotherapy and Transmission Control Databank as of January 15^th^ 2015

^3^ Updated Database, WHO Preventive Chemotherapy and Transmission Control Databank as of April 15^th^ 2015

^4^ Proportion of pre-SAC population requiring deworming for the countries not responding

Of the 73 data points in 39 countries with validated data in the UNICEF deworming database, 24 data points (33%) were excluded from analysis because the number of pre-SAC reached and targeted was considered to exceed plausible estimates of demographic data using WHO criteria. A total of 14 (19%) data points were identical to existing entries in the WHO PCT databank and were thus dropped in favor of their WHO PCT counterparts, while 35 (48%) data points were unique and thus included into the PCT databank. Of these unique data points, 21 (64%) were from 12 countries previously unrepresented in the PCT databank.

Of the 68 points in the initial WHO PCT databank, 14 (21%) data points were identical to points in the UNICEF deworming database and were chosen in favor of their UNICEF deworming database counterparts, 49 (72%) points were included in the updated PCT databank while 5 (7%) points were excluded from analysis as they were replaced with data points from the UNICEF deworming database. The updated databank was comprised of 98 MDAs conducted in 55 countries, of which 62 (63%) were campaign-style events similar to CHDs. The updated databank represents 80.4% of the global pre-SAC population requiring deworming. Africa experienced the biggest coverage increase, from 19.5% to 76.8%. A total of 57 countries requiring deworming are not listed in the PCT databank, leading to a coverage gap of 19.6%.

### Number of Pre-SAC Treated and Coverage

In the initial analysis of the PCT databank, 63.9 million pre-SAC that required deworming received the intervention in 2013 (Table 2). The global coverage estimate in this version of the PCT databank was 23.9%. South-East Asia region (40.2%) had the highest coverage followed by the Americas (33.3%) and Western Pacific Region (22.9%).

**Table 2:** Global coverage of deworming among preschool-age children in the World Health Organization Preventive Chemotherapy and Transmission Control Databank, 2013.

		Initial Database^2^		Updated Database^3^	
Regions^1^	Number of pre-SAC requiring deworming	Number of pre-SAC requiring deworming and treated	Coverage^4^ (%)	Number of pre-SAC requiring deworming and treated	Coverage^4^ (%)
Africa	106,922,486	13,832,969	12.9	52,748,227	49.3
The Americas	13,053,005	4,339,867	33.3	4,339,867	33.3
East Mediterranean	22,520,466	0	0.0	8,337,077	37.0
Europe	324,835	40,099	12.3	40,099	12.3
South-East Asia	100,926,923	40,520,580	40.2	60,005,100	59.5
Western Pacific	22,803,817	5,222,389	22.9	5,268,699	22.9
Global	266,551,532	63,955,904	23.9	130,739,070	49.1

pre-SAC, preschool-age children.

^1^ WHO Regions

^2^ Initial Database, WHO Preventive Chemotherapy and Transmission Control Databank as of January 15^th^ 2015

^3^Updated Database, WHO Preventive Chemotherapy and Transmission Control Databank as of April 15^th^ 2015

^4^Proportion of pre-SAC population requiring deworming that received treatment

**Fig 2:**
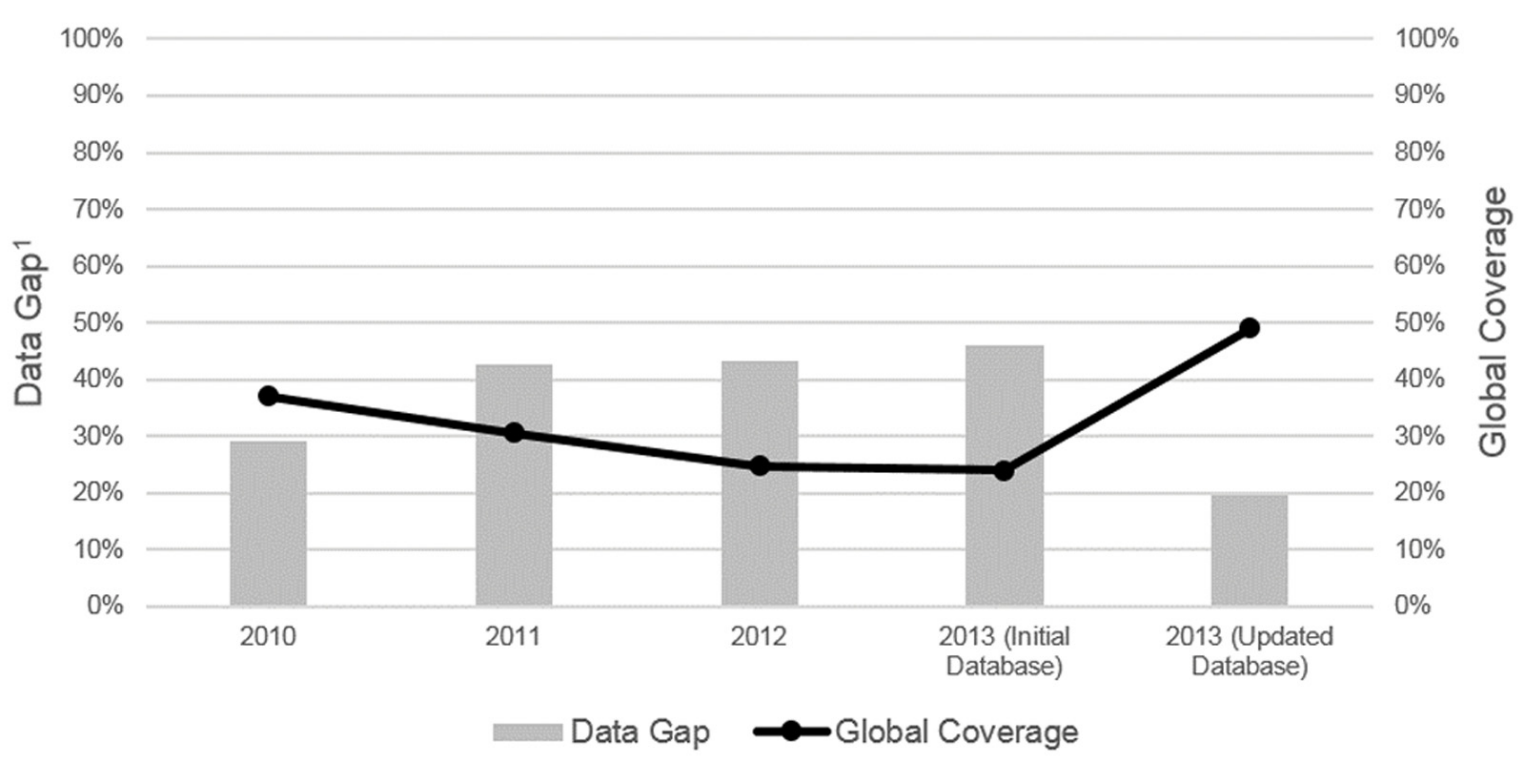
Annual pre-SAC global coverage and data gaps^1^, 2013. Proportion of global pre-SAC population requiring deworming and treated and the global data gap for the years (2010–2013). pre-SAC, preschool-age children. ^1^Data gap is proportion of pre-SAC population requiring deworming for the countries not responding. ^2^Initial Database, WHO Preventive Chemotherapy and Transmission Control Databank as of January 15^th^ 2015, Updated Database ^3^WHO Preventive Chemotherapy and Transmission Control Databank as of April 15^th^ 2015

In the updated databank, the number of pre-SAC requiring deworming who were treated increased to 130.7 million, thus more than doubling the global coverage from 23.9% to 49.1% (Fig 2). The highest increases in pre-SAC treated were observed in Africa and South-East Asia with an additional 41.4 and 19.4 million additional pre-SAC treated, respectively.

**Fig 3:**
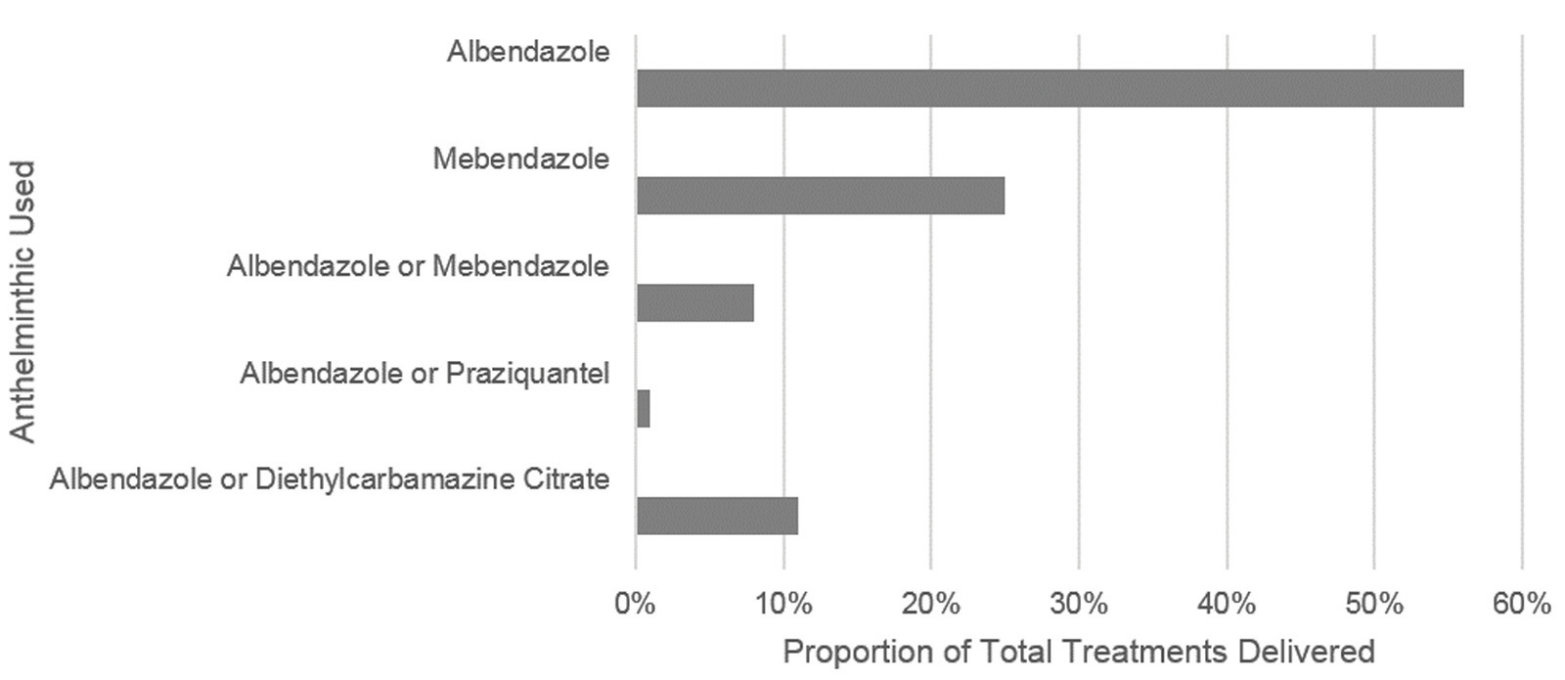
Proportion of total treatments by Anthelminthic, 2013 (updated database). Updated Database, WHO Preventive Chemotherapy and Transmission Control Databank as of April 15^th^ 2015.

With the update of the PCT databank, the number of treatments administered increased from 94.7 million to 234.8 million (Table 3). Of the treatments delivered in 2013 according to the updated PCT databank, albendazole was used exclusively in 39 rounds (56% of all treatments), mebendazole was used exclusively in 26 rounds (25% of all treatments), and a combination of anthelminthics (always including albendazole) in the remaining rounds (Fig 3).

**Table 3:** Number of deworming treatments administered among preschool-age children in the World Health Organization Preventive Chemotherapy and Transmission Control Databank, 2013.

	Initial Database^2^		Updated Database^3^	
Regions^1^	Number of treatments delivered	Proportion of total treatments (%)	Number of treatments delivered	Proportion of total treatments (%)
Africa	23,406,999	24.7	101,870,610	43.4
The Americas	10,580,728	11.2	10,580,728	4.5
East Mediterranean	35,830	0.0	14,900,158	6.3
Europe	995,019	1.1	995,019	0.4
South-East Asia	51,629,744	54.5	98,287,744	41.9
Western Pacific	8,083,362	8.5	8,162,227	3.5
Global	94,731,682	100.0	234,796,486	100.0

^1^ WHO Regions

^2^ Initial Database, WHO Preventive Chemotherapy and Transmission Control Databank as of January 15^th^ 2015

^3^Updated Database, WHO Preventive Chemotherapy and Transmission Control Databank as of April 15^th^ 2015

## Discussion

With the current CHD deworming coverage assessment, a total of 35 new data points were added to the WHO PCT databank. These new data points captured an additional 140.1 million deworming dosages, thus increasing the global number of pre-SAC deworming dosages administered in 2013 to 234.8 million. With this inclusion, the global 2013 pre-SAC deworming coverage increased from 23.9% to 49.1%.

This new global pre-SAC deworming coverage estimate thwarts apparent global coverage decreases observed from 2010 to 2012 [8–10]. In fact, the estimate puts global deworming programs back on track to attain the 50% coverage by 2015 [13]. The update also aligns with the 2012 London Declaration on Neglected Tropical Diseases (NTDs) goals of fostering collaboration and coordination on NTDs and providing regular updates on the progress in reaching 2020 goals and remaining gaps [20]. Nevertheless, given that 57 countries requiring require deworming are not represented in the PCT databank, there is insufficient progress towards demonstrating that all countries requiring deworming start programs by 2015 [6].

CHDs delivered nearly half of all global pre-SAC treatments in 2013, thus illustrating the strategic importance of this delivery mechanism for attaining global pre-SAC coverage goals. CHDs may take the form of integrated special immunization activities or special events designed to deliver VAS and other high-impact interventions among to pre-SAC. The design of CHDs and the package of interventions offered can be tailored to the local contexts [21]. In fragile health systems, CHDs serve as a major delivery platform for high-impact interventions targeted to pre-SAC [22, 23]. In settings with stronger health systems, they can be increasingly integrated into decentralized, routine primary health care, such as through the local budgeting and management of the events by health districts [21]. Nevertheless, CHDs may divert resources away from the delivery of routine health services [14].

In CHDs, deworming is often co-delivered alongside VAS owing to logistical and epidemiological considerations [2, 14]. Deworming may increase the acceptability of other high-impact interventions delivered in CHDs because caretakers consider the excretion of worms in the child’s feces observed shortly after drug administration as a sign of improved child health [24]. Given the frequent co-delivery of VAS and deworming, the global, annual two-dose VAS coverage of 65% may serve as an indication of pre-SAC deworming coverage achievable through co-delivery with VAS [25].

Prior to this coverage assessment, global pre-SAC deworming was inadequately covered in the official 2013 PCT database. This data gap is due to incomplete reporting of CHD activities to WHO Geneva. Similar reporting gaps have been observed previously for deworming medications delivered by non-governmental organizations [26] and for deworming drugs delivered outside of national STH control programs [27]. Ideally, national Ministry of Health NTD focal persons would collect and report deworming coverage to WHO Geneva. As links with NTD focal persons at national levels are strengthened, UNICEF will continue the systematic coverage assessment of deworming delivered to pre-SAC through CHDs.

The global deworming coverage assessment identified 24 data points from integrated poliomyelitis campaigns that also delivered deworming but that could not be included in the PCT databank because the number of pre-SAC reached and targeted exceeded WHO-defined levels deemed to be plausible. If these data points had been accepted, the global number of pre-SAC treated and the total number of treatments delivered would have increased by an additional 27.7 and 99.2 million, respectively. This inclusion would have increased the global pre-SAC coverage to 59.1%. In subsequent global coverage reporting exercises, harmonization of data rules employed by the STH- and poliomyelitis communities is warranted. As progress is maintained towards the eradication of poliomyelitis, the number of supplementary immunization activities delivering deworming and other high-impact interventions will decrease substantially [28]. As a result, national health planners need to ensure that STH control is reflected in the poliomyelitis transition plans and explore other delivery mechanism for VAS, deworming, and other child interventions currently delivered through vertical CHD-type events.

This coverage assessment is limited by a number of factors. First, the CHD deworming coverage data collected is generally based on tally sheets completed during the supplementation events. Numerators generated from tally sheets may suffer from discounting, double counting or summation errors during their aggregation [29]. However, this system has been a long-standing data source for the global tracking of VAS coverage trends, and is deemed to be useful for the tracking deworming coverage trends as well [15]. Second, the district-disaggregated demographic and epidemiologic data used to generate estimates of children requiring deworming have limited accuracy, especially when sanitation data are used as a proxy for epidemiologic data [30]. Third, the current assessment focused on the 82 global VAS priority countries, and thus did not cover 35 countries requiring deworming. The scope of the coverage assessment will be extended to all global deworming priority countries in the future and to increase response rates among all countries contacted. Fourth, the link between pre-SAC deworming coverage data as presented here needs to be more rigorously linked to assessments demonstrating health impact analyses, and lastly, more information is needed on the quality of deworming formulations used in MDA programs [2].

In sum, this CHD coverage assessment showed that global pre-SAC deworming programs achieved more than double the coverage previously reported, and are therefore on track to achieve 2015 global pre-SAC coverage targets. With the increased attention on the role of NTDs for equitable and sustainable development [31, 32], deworming coverage reporting and program delivery should be further strengthened in an effort to reach pre-SAC in greatest need and to achieve the global 75% coverage target for 2020.
